# Supplementary material for: A mutualistic interaction between Streptomyces bacteria, strawberry plants and pollinating bees
Source: Nat Commun. 2019 Oct 22;10:4802. doi: 10.1038/s41467-019-12785-3 (PMC6805876; doi:10.1038/s41467-019-12785-3)
Supplement: Supplementary file 1 — Supplementary information [file 41467_2019_12785_MOESM1_ESM.pdf]

Supplementary Information

A mutualistic interaction between *Streptomyces* bacteria, strawberry plants and pollinating bees

Kim *et al.*

Supplementary Table 1. Number of sequencing read counts of strawberry flower and strawberry pollen samples

Sources	Sample	Number of reads	Total bases
Strawberry flowers	0 week	6,113	2,334,047
	2 week	49,097	19,299,264
	4 week	35,738	14,681,860
	6 week	53,839	21,708,171
	8 week	31,980	12,959,159
	10 week	16,734	6,281,326
	12 week	45,863	18,041,976
	14 week	53,673	22,253,896
	16 week	51,456	21,294,631
	18 week	57,480	23,779,210
	20 week	41,952	17,420,498
	22 week	52,955	21,897,136
	24 week	50,999	21,404,489
Pollen from the bodies of bees	2 week	24,658	9,751,433
	4 week	25,350	9,814,156
	6 week	14,854	5,682,473
	8 week	43,839	17,410,702
	10 week	37,530	13,883,101
	16 week	58,003	23,471,585
	18 week	66,162	27,650,600
	20 week	64,467	26,798,108
	22 week	50,898	21,022,749
Total		933,640	378,840,570

Supplementary Table 2. Sorted data of sequencing libraries from samples of strawberry flower and pollen from the bodies of bees. Samples were collected from November 2013 to March 2014

Status	Reads	Total bases
Sorted	933,640	378,840,570
ERR[a]	5	2,167
NA[b]	80,446	23,811,829
LowQual[c]	50	-

[a]ERR: with a different barcode sequence on both ends

[b]NA: without a barcode sequence

[c]LowQual: length=0 (after trimming barcode sequence)

Supplementary Table 3. Fifteen most abundant OTUs on strawberry flowers during high diversity (HD) periods compared to low diversity (LD) periods

Phylum	Family	Representative OTU		
		Strain	Silva accession number	Number of reads
Proteobacteria	Pseudomonadaceae	*Pseudomonas* sp.	GU476606	11939
	Alcaligenaceae	uncultured bacterium	AB193943	20421
	Moraxellaceae	*Acinetobacter baumannii* AB0057	CP001182	12132
	Methylobacteriaceae	*Methylobacterium* sp. CBMB130	AY683048	720
	Alcaligenaceae	uncultured *Lautropia* sp.	DQ016725	350
	Comamonadaceae	uncultured bacterium	FJ894546	216
	Neisseriaceae	uncultured bacterium	EF511167	196
	Xanthomonadaceae	*Xanthomonas fragariae* LMG 708	X95920	123
	Alcaligenaceae	uncultured bacterium	GQ264057	344
Actinobacteria	Streptomycetaceae	*Streptomyces globisporus* subsp. *globisporus* NRRL B-2872	EF178686	1479
	Propionibacteriaceae	uncultured bacterium	GQ083339	448
Firmicutes	Streptococcaceae	*Streptococcus sanguinis* SK36	CP000387	705
	Staphylococcaceae	uncultured bacterium	GQ028474	308
	Streptococcaceae	uncultured Firmicutes bacterium	EF682877	218
	Staphylococcaceae	uncultured bacterium	GQ095668	242

Supplementary Table 4. Fifteen most abundant OTUs during high diversity (HD) periods as compared to low diversity (LD) periods in pollen

Phylum	Family	Representative OTU		
		Strain	Silva accession number	Number of reads
Proteobacteria	Bartonellaceae	uncultured alpha proteobacterium	DQ837624	1002
	Alcaligenaceae	uncultured bacterium	AB193943	17962
	Burkholderiaceae	*Ralstonia pickettii* 12D	CP001644	6138
	Neisseriaceae	uncultured *Simonsiella* sp.	AY370189	1537
	Bartonellaceae	uncultured alpha proteobacterium	DQ837624	1002
	Alcaligenaceae	uncultured *Lautropia* sp.	DQ016725	1375
Actinobacteria	Propionibacteriaceae	uncultured bacterium	GQ083339	3118
	Streptomycetaceae	*Streptomyces globisporus* subsp. *globisporus* NRRL B-2872	EF178686	1927
Firmicutes	Lactobacillaceae	uncultured Firmicutes bacterium	DQ837637	2303
	Lactobacillaceae	uncultured *Lactobacillus* sp.	HM046579	1244

Supplementary Table 5. Common microbial taxa on strawberry flowers (SF) and strawberry pollen (SP) among the top 15 OTUs

Flower and pollen sample	Diversity	Family	Number of reads	Abundance (%)
2 week	HD	Moraxellaceae	44	65.67
		Bartonellaceae	23	34.33
4 week	HD	Moraxellaceae	52	83.87
		Streptomycetaceae	10	16.13
6 week	HD	Pseudomonadaceae	41	56.56
		Streptomycetaceae	44	48.9
		Alcaligenaceae	5	5.56
8 week	HD	Pseudomonadaceae	72	52.17
		Streptomycetaceae	66	47.83
10 week	HD	Oxalobacteraceae	533	81.71
		Alcaligenaceae	87	13.34
		Streptomycetaceae	23	3.53
		Methylobacteriaceae	9	1.38
16 week	LD	Pseudomonadaceae	50	94.33
		Enterobacteriaceae	2	3.77
		Bradyrhizobiaceae	1	1.87
18 week	LD	Pseudomonadaceae	1883	99.69
		Enterobacteriaceae	4	0.22
		Oxalobacteraceae	2	0.11
20 week	LD	Pseudomonadaceae	1975	100
22 week	LD	Pseudomonadaceae	1932	98.78
		Bradyrhizobiaceae	17	0.87
		Sphingomonadaceae	5	0.25
		Oxalobacteraceae	2	0.1

Supplementary Table 6. Abundance (%) and number of reads of common OTUs in the low disease period

Phylum	OTU %		Number of reads	
	SF	SP	SF	SP
Actinobacteria	44.91	43.85	890	2912
Proteobacteria	20.75	25.31	1927	5045
Firmicutes	34.34	30.83	1473	3547

Supplementary Table 7. Isolates and screening of antifungal bacteria from flowers against *Botrytis cinerea*

	0 week	2 week	4 week	6 week	8 week	10 week	12 week	14 week	16 week	18 week	20 week	22 week	24 week	Total
No. of isolates	32	78	60	94	42	67	83	35	49	88	84	95	80	887
1st screen	3	6	0	7	1	1	58	8	2	12	2	1	15	116
2nd screen	0	4	0	3	1	1	58	3	0	4	1	0	3	78
3rd screen	0	0	0	2	0	0	39	0	0	2	1	0	0	44

Supplementary Table 8. Isolates and screening of antifungal strains from pollen against *Botrytis cinerea*

	2 week	4 week	6 week	8 week	10 week	16 week	18 week	20 week	22 week	Total
No. of isolates	13	31	24	32	22	44	45	68	45	324
1st screen	0	1	0	0	12	6	10	6	3	38
2nd screen	0	1	0	0	8	6	7	5	3	30
3rd screen	0	1	0	0	8	2	4	4	3	22

Supplementary Table 9. Characteristics of antimicrobial *Streptomyces* from flowers and pollen

Origin	Isolate	Sporulation activity	*Botrytis cinerea*	*Serratia marcescens*	*Paenibacillus larvae*	16S RNA identification (%)
Flower	SF7B6	++	+++	++++	+++	*Streptomyces globisporus* (99%)<br>*Streptomyces badius* (99%)
	SF7C7	++++	+++	+++	-	*Streptomyces globisporus* (99%)<br>*Streptomyces badius* (99%)
	SF7C9	++	+++	++	++	*Streptomyces globisporus* (99%)<br>*Streptomyces griseus* (99%)
	SF7C11	++	+++	+++	+	*Streptomyces globisporus* (99%)<br>*Streptomyces badius* (99%)
	SF7E3	++++	+++	++	+	*Streptomyces globisporus* (99%)<br>*Streptomyces griseus* (99%)
	SF7F2	++	+++	+++	+++	*Streptomyces globisporus* (99%)<br>*Streptomyces griseus* (99%)
	SF7F6	+++	+++	+++	++	*Streptomyces globisporus* (99%)<br>*Streptomyces griseus* (99%)
Pollen	SP6C4	+++	+++	++++	+++	*Streptomyces globisporus* (99%)<br>*Streptomyces badius* (99%)
	SP6E4	+++	++	-	+	*Streptomyces globisporus* (99%)<br>*Streptomyces griseus* (99%)
	SP6F4	++	++	+++	++	*Streptomyces globisporus* (99%)<br>*Streptomyces griseus* (99%)
	SP6G4	++	++	+++	+++	*Streptomyces globisporus* (99%)<br>*Streptomyces griseus* (99%)
	SP6G8	++	++	++	+++	*Streptomyces globisporus* (99%)<br>*Streptomyces griseus* (99%)
	SP9F2	+	+++	++	-	*Streptomyces globisporus* (99%)<br>*Streptomyces badius* (99%)
	SP12C6	+	+++	++	+++	*Streptomyces globisporus* (99%)<br>*Streptomyces badius* (99%)
	SP12C7	++	++	++	+++	*Streptomyces globisporus* (99%)<br>*Streptomyces badius* (99%)
	SP12F4	+++	++	++	++	*Streptomyces globisporus* (99%)<br>*Streptomyces badius* (99%)

++++: the highest inhibition (clear zone**,** > 1.5 cm)

+++: strong inhibition (1 cm ≤ clear zone < 1.5 cm)

++: medium inhibition (0.5 cm ≤ clear zone < 1 cm)

+: low inhibition (0.1 cm ≤ clear zone < 0.5 cm)

-: no inhibition (no clear zone)

Supplementary Table 10. Number of sequencing read counts of strain SP6C4 sprayed and delivered by bee-vectoring samples

	Source	Sample	Number of reads	Total bases
Greenhouse A (January 2016 to March 2016)	Strawberry flower (SP6C4 - sprayed)	0 Week	543,190	89,754,932
		2 week	553,030	131,761,165
		4 week	345,214	141,225,982
		6 week	341,550	102,063,755
		8 week	365,814	109,022,282
		10 week	361,860	107,920,825
	Strawberry flower (Control for spray)	0 week	299,786	89,754,935
		2 week	442,158	131,761,165
		4 week	475,692	141,225,982
		6 week	447,956	141,225,982
		8 week	349,530	104,628,552
		10 week	482,114	143,628,552
Greenhouse B (November 2016 to February 2017)	Strawberry flower (SP6C4-delived by bee-vectoring)	0 week	146,525	67,442,170
		2 week	207,574	93,871,154
		4 week	152,768	69,810,669
		6 week	141,087	65,068,718
		8 week	142,619	65,593,241
		10 week	172,769	77,781,223
Greenhouse C (November 2016 to February 2017)	Strawberry flower (Control for bee-vectoring)	0 week	130,712	60,363,470
		2 week	187,039	83,562,213
		4 week	197,211	90,688,410
		6 week	186,011	85,198,029
		8 week	152,346	68,996,441
		10 week	207,890	93,483,592
Total			7,032,445	2,335,833,439

Supplementary Table 11. Sorted data from sequencing libraries of SP6C4 sprayed and delivered by bee-vectoring samples

Status	Reads	Total bases
Sorted	7,032,445	2,335,833,439
ERR[a]	3	1,793
NA[b]	18,507	3,797,278
LowQual[c]	50	-

[a]ERR: with a different barcode sequence on both ends

[b]NA: without a barcode sequence

[c]LowQual: length=0 (after trimming barcode sequence)

Supplementary Table 12. GenBank accession numbers for pyrosequencing and genome sequencing

	Flower (2013 to 2104)	Pollen (2013 to 2014)	SP6C4 sprayed flower	SP6C4 control flower (sprayed)	SP6C4-delivered flower by bee-vectoring	SP6C4 control flower (bee-vectoring)	S4-7 genome	SF7B6 genome	SP6C4 genome
SAR	SRP150491	SRP150494	SRR9650646	SRR9650684	SRP150493	SRP150495	SRP151194	SRP151167	SRP151166
BioProject	PRJNA392201	PRJNA392205	PRJNA552620	PRJNA552619	PRJNA416688	PRJNA416686	PRJNA237995	PRJNA316661	PRJNA316658
BioSample	SAMN07962168	SAMN07962169	SAMN12217317	SAMN12217318	SAMN07962165	SAMN07962161	SAMN03320103	SAMN04589202	SAMN04589131
Genome	n/a	n/a	n/a	n/a	n/a	n/a	JYBE00000000.2	LWMP00000000	LWMQ00000000

Supplementary Table 13. List of gene primer sets for identification of bacteria

Gene	Primer sequence (5’ – 3’)	Size (bp)	Reference
gyrB PF	GAGGTCGTGCTGACCGTGCTGCACGCGGGCGGCAAGTTCGGC	1305	44
gyrB PR	GTTGATGTGCTGGCCGTCGACGTCGGCGTCCGCCAT		
rec APF	CCGCRCTCGCACAGATTGAACGSCAATTC	913	44
rec APR	GCSAGGTCGGGGTTGTCCTTSAGGAAGTTGCG		
16S rRNA-27F	AGAGTTTGATCMTGGCTCAG	1400	45
16S rRNA-1492R	TACGGYTACCTTGTTACGACTT		

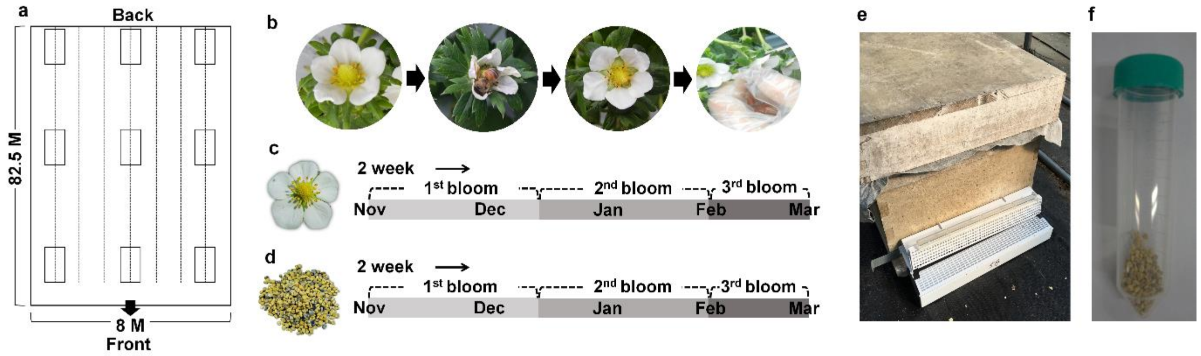

Supplementary Figure 1. Flower and pollen collection in the strawberry greenhouse. **a,** Plot design used to collect flower samples. In the greenhouse (W: 8 M and H: 82.5 M, area: 660 m^2), three areas within three rows of plants were sampled. Each area had three zones with a length of 5 meters. **b,** Illustration of flower collection. **c**,**d,** During flower and pollen collection periods, samples were collected every other week. Strawberry plants blossomed three times (1st, 2nd and 3rd) during the sampling period. **e,** Pollen collector and, **f** collected pollen in a 50-mL Falcon tube.

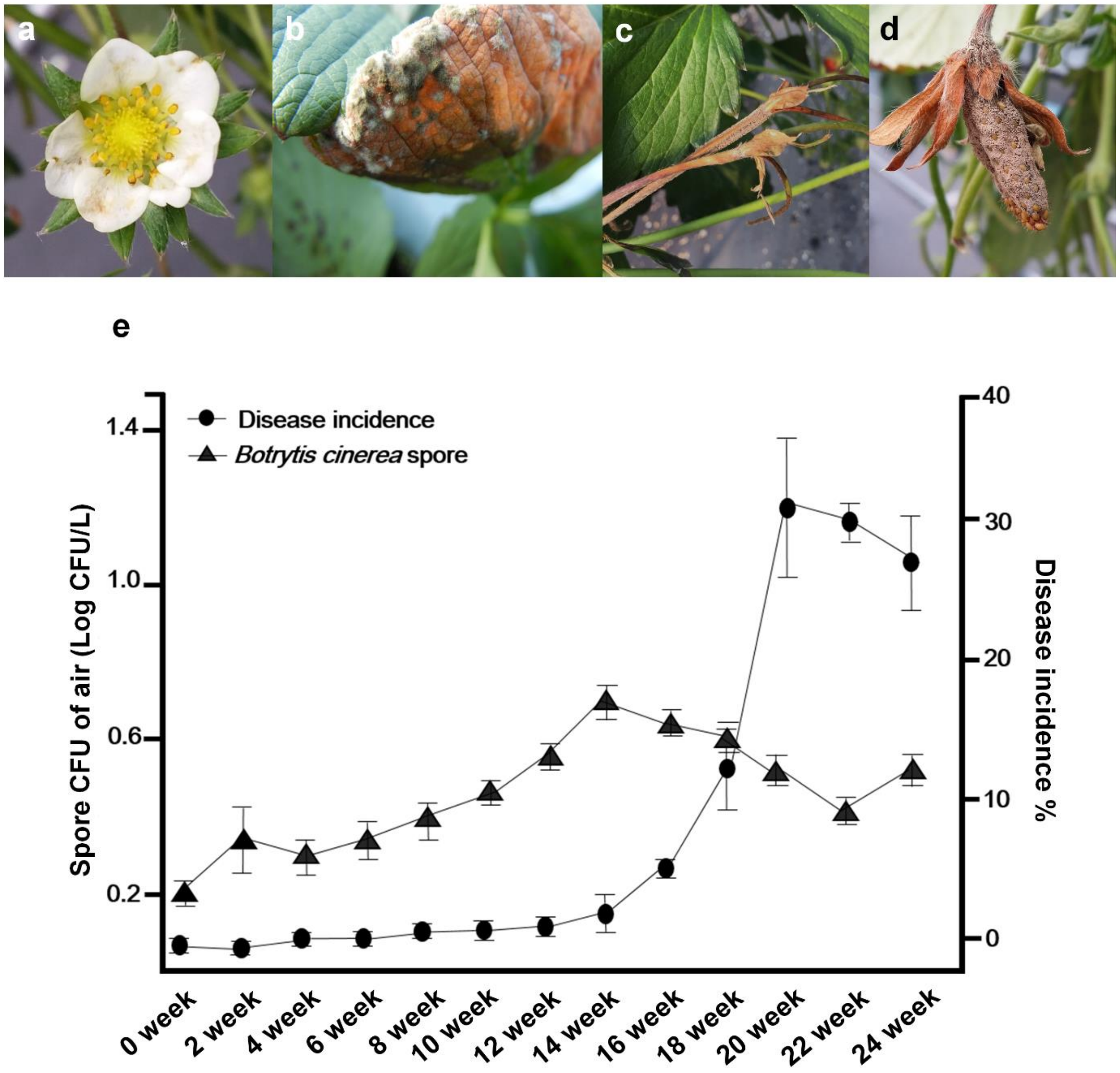

Supplementary Figure 2. Gray mold disease incidence and conidia concentration in a strawberry greenhouse. Symptoms and signs of strawberry gray mold disease **a,** water soaked and brown spots on flower petals, **b,** gray mold hyphae and conidia covering the leaf, **c,** browning of stems, **d,** mummification of berry fruits and conidia-covered berries. **e,** Relationship between conidial concentration and gray mold disease incidence in the strawberry greenhouse (closed circle). *B. cinerea* conidia were captured with a spore collector, germinated, and hyphal growth on BSTM media was used to calculate conidia density (closed triangle).

Bars represent standard error of nine blocks, each block contained 150 plants.

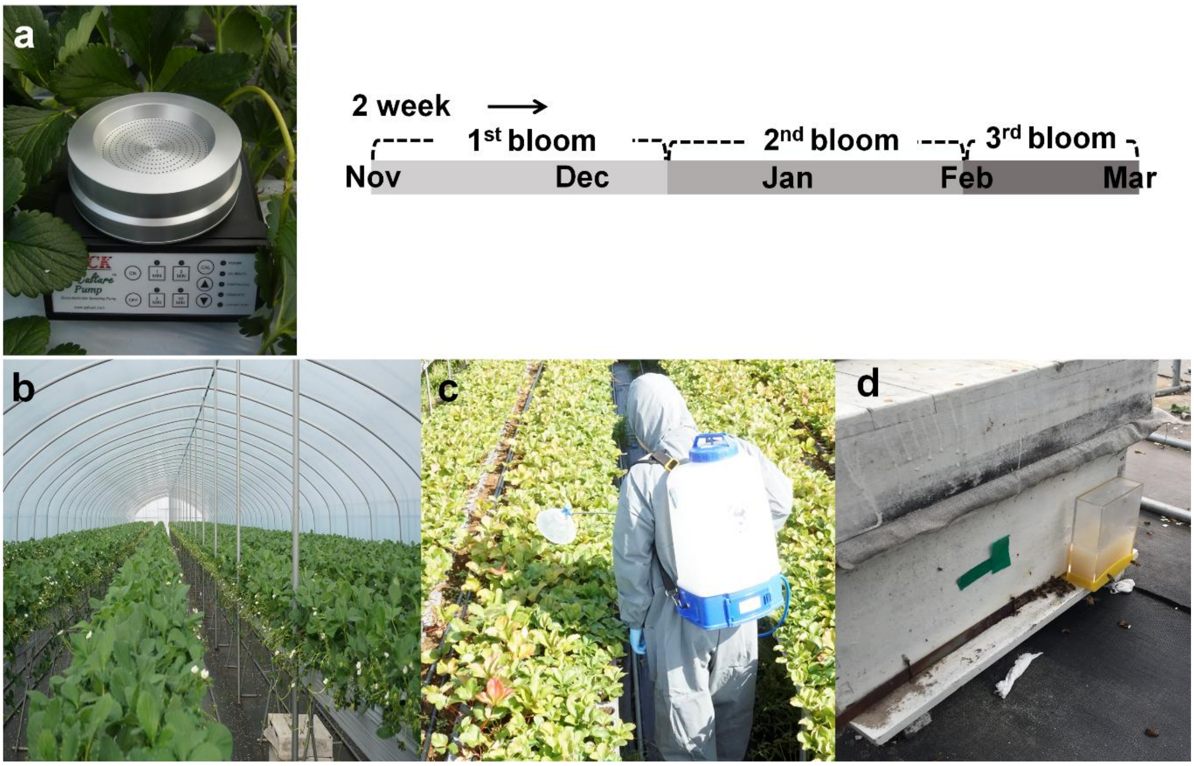

Supplementary Figure 3. *Botrytis cinerea* spore collection and greenhouse applications of SP6C4 strain. **a,** *B. cinerea* spores were collected with a spore trap every other week. **b** and **c,** a greenhouse with SP6C4 sprayed or not. The bacteria (10^7 cfu/mL with 0.1% methyl cellulose) were applied by a sprayer (HP-2010, Korea, 1.5 L discharge capacity/min). Sprayed and control blocks had 3 replications (n = 450 plants per block). **d,** a bee-vectoring device at the bee passageway. The device holds SP6C4 (10^7 cfu/mL) in 12.5% PEG, 3% skim milk.

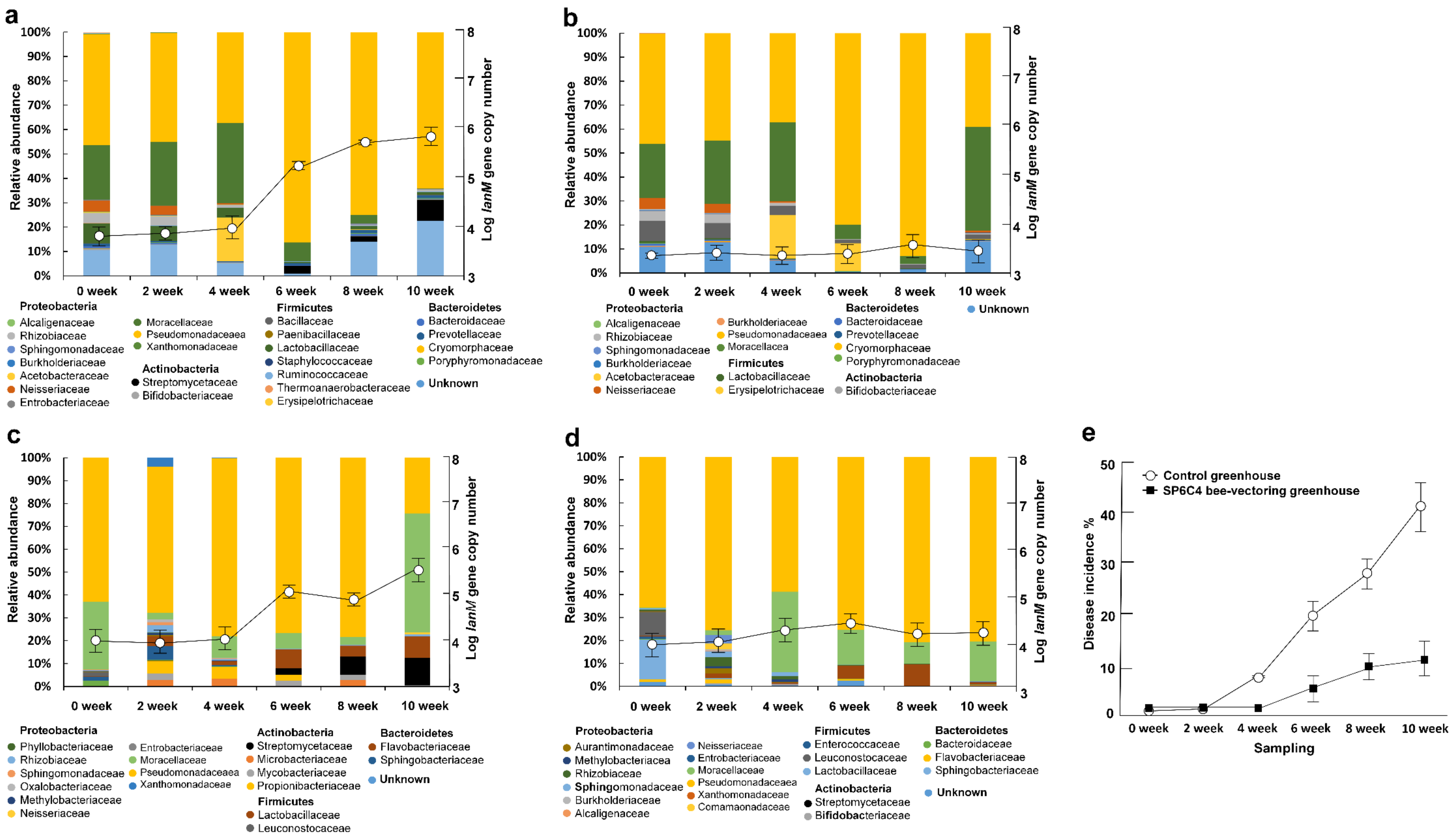
a
b
c
d
e
Relative abundance
Log lanM gene copy number
0 week
2 week
4 week
6 week
8 week
10 week
Proteobacteria
Alcaligenaceae
Rhizobiaceae
Sphingomonadaceae
Burkholderiaceae
Acetobacteraceae
Neisseriaceae
Entrobacteriaceae
Moracellaceae
Pseudomonadaceaea
Xanthomonadaceae
Actinobacteria
Streptomycetaceae
Bifidobacteriaceae
Firmicutes
Bacillaceae
Paenibacillaceae
Lactobacillaceae
Staphylococcaceae
Ruminococcaceae
Thermoanaerobacteraceae
Erysipelotrichaceae
Bacteroidetes
Bacteroidaceae
Prevotellaceae
Cryomorphaceae
Poryphyromonadaceae
Unknown
Proteobacteria
Alcaligenaceae
Rhizobiaceae
Sphingomonadaceae
Burkholderiaceae
Acetobacteraceae
Neisseriaceae
Burkholderiaceae
Pseudomonadaceaea
Moracellacea
Firmicutes
Lactobacillaceae
Erysipelotrichaceae
Bacteroidetes
Bacteroidaceae
Prevotellaceae
Cryomorphaceae
Poryphyromonadaceae
Actinobacteria
Bifidobacteriaceae
Unknown
Proteobacteria
Phyllobacteriaceae
Rhizobiaceae
Sphingomonadaceae
Oxalobacteriaceae
Methylobacteriaceae
Neisseriaceae
Entrobacteriaceae
Moracellaceae
Pseudomonadaceaea
Xanthomonadaceae
Actinobacteria
Streptomycetaceae
Microbacteriaceae
Mycobacteriaceae
Propionibacteriaceae
Firmicutes
Lactobacillaceae
Leuconostocaceae
Bacteroidetes
Flavobacteriaceae
Sphingobacteriaceae
Unknown
Proteobacteria
Aurantimonadaceae
Methylobacteriacea
Rhizobiaceae
Sphingomonadaceae
Burkholderiaceae
Alcaligenaceae
Neisseriaceae
Entrobacteriaceae
Moracellaceae
Pseudomonadaceaea
Xanthomonadaceae
Comamaonadaceae
Firmicutes
Enterococcaceae
Leuconostocaceae
Lactobacillaceae
Actinobacteria
Streptomycetaceae
Bifidobacteriaceae
Bacteroidetes
Bacteroidaceae
Flavobacteriaceae
Sphingobacteriaceae
Unknown
Control greenhouse
SP6C4 bee-vectoring greenhouse
Disease incidence %
Sampling

Supplementary Figure 4. Pyrosequencing of microbes from flowers and *Streptomyces* density detection with *lanM* gene by qPCR in SP6C4 applied by spraying or vectored by bees. **a-d,** Microbial community and *lanM* gene copy numbers. **a,** sprayed SP6C4 (10^7 cfu/mL). **b,** control for spraying. **c,** SP6C4 delivered by bees-vectoring. **d,** control for bee-vectoring. Line graphs represent *Streptomyces* density determined by *lanM* gene copy number. **e**, gray mold disease incidence in plots with SP6C4 delivered by bee-vectoring and control. Bars represent standard error. **a-d** Source data are provided as a Source Data file.
