# Supplementary material for: A mutualistic interaction between Streptomyces bacteria, strawberry plants and pollinating bees
Source: Nat Commun. 2019 Oct 22;10:4802. doi: 10.1038/s41467-019-12785-3 (PMC6805876; doi:10.1038/s41467-019-12785-3)
Supplement: Supplementary file 3 — Reporting Summary [file 41467_2019_12785_MOESM3_ESM.pdf]

nature research

Corresponding author(s): Youn-Sig Kwak

Last updated by author(s): Sep 20, 2019

Reporting Summary

Nature Research wishes to improve the reproducibility of the work that we publish. This form provides structure for consistency and transparency in reporting. For further information on Nature Research policies, see Authors & Referees and the Editorial Policy Checklist.

Statistics

For all statistical analyses, confirm that the following items are present in the figure legend, table legend, main text, or Methods section.

n/a	Confirmed	
☐	☒	The exact sample size (*n*) for each experimental group/condition, given as a discrete number and unit of measurement
☐	☒	A statement on whether measurements were taken from distinct samples or whether the same sample was measured repeatedly
☐	☒	The statistical test(s) used AND whether they are one- or two-sided<br>*Only common tests should be described solely by name; describe more complex techniques in the Methods section.*
☐	☒	A description of all covariates tested
☐	☒	A description of any assumptions or corrections, such as tests of normality and adjustment for multiple comparisons
☐	☒	A full description of the statistical parameters including central tendency (e.g. means) or other basic estimates (e.g. regression coefficient) AND variation (e.g. standard deviation) or associated estimates of uncertainty (e.g. confidence intervals)
☐	☒	For null hypothesis testing, the test statistic (e.g. *F*, *t*, *r*) with confidence intervals, effect sizes, degrees of freedom and *P* value noted<br>*Give P values as exact values whenever suitable.*
☒	☐	For Bayesian analysis, information on the choice of priors and Markov chain Monte Carlo settings
☒	☐	For hierarchical and complex designs, identification of the appropriate level for tests and full reporting of outcomes
☒	☐	Estimates of effect sizes (e.g. Cohen's *d*, Pearson's *r*), indicating how they were calculated

Our web collection on statistics for biologists contains articles on many of the points above.

Software and code

Policy information about availability of computer code

Data collection	Pyrosequencing: a Roche Genome Sequencer (GS) FLX software (v 3.0)<br>Genome: single molecule real time (SMRT: https://www.pacb.com/smrt-science/smrt-sequencing/) and non-hybrid hierarchical genome (HGAP version 2.3)
Data analysis	Pyrosequencing: QIIME (http://qiime.org/)<br>Genome: pre-assembly Quiver (http://www.pacificbiosciences.com/devnet/),the Web ACT program (Artemis Comparison Tool, Center for Bioinformatics), BLAST Ring Image Generator (BRIG) v 0.95 (http://sourceforge.net/projects/brig/), RAST version 2.0, antiSMASH (http://antismash.secondarymetabolites.org) version 3.0.5<br>Comparative genomics: the Web ACT program (Artemis Comparison Tool, Center for Bioinformatics). BLAST Ring Image Generator (BRIG) v 0.95 (http://sourceforge.net/projects/brig/)<br>others: SIGMA PLOT ver. 11.0

For manuscripts utilizing custom algorithms or software that are central to the research but not yet described in published literature, software must be made available to editors/reviewers. We strongly encourage code deposition in a community repository (e.g. GitHub). See the Nature Research guidelines for submitting code & software for further information.

Data

Policy information about availability of data

All manuscripts must include a data availability statement. This statement should provide the following information, where applicable:

- Accession codes, unique identifiers, or web links for publicly available datasets
- A list of figures that have associated raw data
- A description of any restrictions on data availability

Sequencing data that support the findings of this study have been deposited in GenBank with the accession codes listed in Supplementary Table 12.

Field-specific reporting

Please select the one below that is the best fit for your research. If you are not sure, read the appropriate sections before making your selection.

☒ Life sciences ☐ Behavioural & social sciences ☐ Ecological, evolutionary & environmental sciences

For a reference copy of the document with all sections, see nature.com/documents/nr-reporting-summary-flat.pdf

Life sciences study design

All studies must disclose on these points even when the disclosure is negative.

Sample size	pyrosequencing: 13 flower samples and 9 pollen samples (year 2013 - 2014); 6 SP6C4-sprayed and 6 nu-sprayed (year 2016); 6 SP6C4-delived by bee-vectering and 6 non-treated samples (year 2016 - 2017).<br>Gray mold disease incidence and spore collection : 13 samples (year 2013 - 2014); 6 samples (Spray, year 2016); 6 samples (Bee-vectoring, year 2016 - 2017)<br>Movement assay: 3 plants and 5 pieces for each plants
Data exclusions	No data were excluded
Replication	Field experiments were conducted with 9 biological replications and other experiments were conducted with 3 or more biological replication.
Randomization	Randomized block design
Blinding	Blinding was not relevant to this study. All samples were collected and analyzed from treated and untreated greenhouse throughout the whole growing season of the plants

Reporting for specific materials, systems and methods

We require information from authors about some types of materials, experimental systems and methods used in many studies. Here, indicate whether each material, system or method listed is relevant to your study. If you are not sure if a list item applies to your research, read the appropriate section before selecting a response.

Materials & experimental systems

n/a	Involved in the study	
☒	☐	Antibodies
☒	☐	Eukaryotic cell lines
☒	☐	Palaeontology
☐	☒	Animals and other organisms
☒	☐	Human research participants
☒	☐	Clinical data

Methods

n/a	Involved in the study	
☒	☐	ChIP-seq
☒	☐	Flow cytometry
☒	☐	MRI-based neuroimaging

Animals and other organisms

Policy information about studies involving animals; ARRIVE guidelines recommended for reporting animal research

Laboratory animals	Honeybee (Apis nearctica), Bumble bee (Bumbus impatiens)
Wild animals	none
Field-collected samples	Botrytis cinerea spore sampling protocol is described in the Methods. All samples were autoclaved at the termination of the experiments.
Ethics oversight	No ethical approval is required

Note that full information on the approval of the study protocol must also be provided in the manuscript.
